# Photo-CIDNP of Solvent-Exposed Flavins in Flavoproteins

**DOI:** 10.1021/acs.jpclett.5c01239

**Published:** 2025-07-02

**Authors:** Anton Schmidt, Hannah Schneider, Boris Illarionov, Adelbert Bacher, Markus Fischer, Stefan Weber

**Affiliations:** † Institute of Physical Chemistry, 9174 University of Freiburg, 79104 Freiburg, Germany; ‡ Hamburg School of Food Science, Institute of Food Chemistry, University of Hamburg, 20146 Hamburg, Germany; ¶ TUM School of Natural Sciences, 85748 Garching, Germany

## Abstract

Photochemically induced dynamic nuclear polarization (photo-CIDNP) is a hyperpolarization NMR technique that enhances the resonances of molecules involved in the formation of spin-correlated radical pairs. In this contribution, the method is used to selectively enhance the resonances of solvent-exposed, protein-bound flavins by adding an electron donor (tryptophan) to the sample prior to irradiation. To the best of our knowledge, this method has not been used in this way before. By applying photo-CIDNP to two different flavoproteins with solvent-exposed flavins, namely flavodoxin A from *Escherichia coli* and lumazine protein (LumP) from *Photobacterium leiognathi* in complex with riboflavin (riboflavin-LumP), we investigate the requirements for radical pair formation. The results reveal that only riboflavin-LumP shows an observable photo-CIDNP effect. Using continuous-wave photo-CIDNP on ^1^H and ^13^C nuclei, flavin resonances can be selectively hyperpolarized. Signal assignment is possible by comparing hyperfine data from time-resolved photo-CIDNP and density functional theory (DFT). In addition, the anionic riboflavin radical is determined as the radical present in the geminate radical pair.

## Graphical abstract

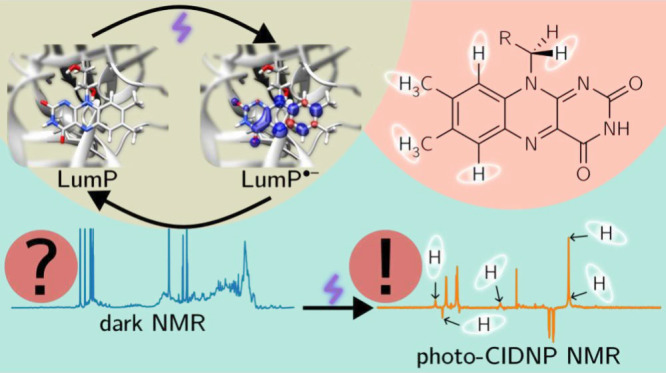

Flavins form a class of cofactors with a 7,8-dimethyl isoalloxazine core capable of absorbing visible light. The widespread occurrence of flavin-binding proteins (flavoproteins) in nature is due to the ability of the redox-active 7,8-dimethyl isoalloxazine ring system to mediate not only two-electron-transfer reactions, but also one-electron-transfer reactions.^1^ The oxidized flavin can be converted to the semiquinone form by one-electron reduction. This one-electron reduced semiquinone form usually occurs either as an anionic radical or as a neutral, protonated radical, depending on the local environment of the flavin.^2,3^ Two-electron reduction of the flavin results in the fully reduced form. Utilizing these three redox states, flavoproteins are involved in various processes, including mediation of numerous redox reactions,^4–7^ and light-dependent processes such as the repair of UV-damaged DNA,^8^ regulation of the circadian timing^9^ and phototropism.^10^

The flavoprotein flavodoxin A (Fld) noncovalently binds flavin mononucleotide (FMN) as chromophore. In cyanobacteria and some algae, Fld acts as a substitute for ferredoxin under iron-limiting conditions.^11^ Both Fld and ferredoxin act as one-electron carriers and are reduced by the terminal acceptors of photosystem I.^12^ In Fld, the protonated semiquinone radical FMNH^•^ is relatively stable.^13^ While the semiquinone radical is formed upon irradiation in the presence of EDTA,^3,14^ optical excitation without a reducing agent does not lead to a long-lived semiquinone radical. Instead, rapid relaxation of the excited singlet state is induced by the formation of a transient radical pair comprising the flavin cofactor and a nearby tryptophan residue.^15^ Recombination of this radical pair regenerates the ground state, thus effectively quenching the photoexcited singlet state on a picosecond time scale.^15^

Riboflavin-LumP behaves very differently upon photoexcitation. After reaching the excited singlet state, intersystem crossing populates the triplet state.^16^ Besides the triplet, minor amounts of an anionic riboflavin radical were observed using transient absorption (TA) spectroscopy. The role of this radical is not yet understood. LumP is also capable of binding 6,7-dimethyl-8-ribityllumazine (DMRL),^17^ with the dissociation constant *K*_d_ of riboflavin being approximately twice that of DMRL.^18^ In contrast to riboflavin-LumP, LumP binding DMRL (DMRL-LumP) shows a rapid decay of the excited singlet state after photoexcitation, which means that no triplet state was observed in these TA spectroscopy measurements.^16^

The oxygen-dependent bioluminescence reaction occurring with DMRL-LumP involves a luciferase, a fatty acid reduction complex and a flavin reductase.^19^ The substrate for the luciferase-catalyzed oxidation of long-chain aliphatic aldehydes is provided by a fatty acid reduction complex consisting of three enzymes.^20^ A flavin reductase yields the reduced FMN which is necessary for the luciferase reaction.^21^ The luciferase reaction involves an excited flavin-4a-hydroxide intermediate, which decays to the ground state by emitting blue light.^22^ However, when DMRL-LumP is present, the energy of the excited state can be transferred to DMRL.^23^ Blue light is then emitted from LumP, although with a bathochromic shift of the emission maximum as compared to that of luciferase. In contrast, this energy transfer does not occur with riboflavin-LumP.^18^ Therefore, the bioluminescence spectrum observed in *Photobacterium* after photoexcitation corresponds to that of DMRL-LumP.^17^ The role of riboflavin-LumP has been hypothesized to be either as a solubility improvement protein for riboflavin or a storage protein for riboflavin, which would otherwise diffuse through the cell walls.^18^

Photo-CIDNP is an NMR hyperpolarization technique that probes nuclear spin polarization arising from reactions involving transient radical pairs.^24^ The method has proven itself both for the detection of radical pairs and for hyperpolarization purposes. Continuous-wave (cw) photo-CIDNP experiments use continuous illumination, while a pulsed laser is used in time-resolved (tr) photo-CIDNP. Continuous illumination has the advantage of stronger signal enhancement, but tr-photo-CIDNP can be used to obtain information about the electronic structure of the radicals forming the radical pair.^25,26^ Photo-CIDNP is often used to study the aromatic amino acids tryptophan, tyrosine and histidine in proteins, since they can act as electron donors in radical pair reactions.^27^ By adding electron acceptors, typically dyes such as flavins^28^ or fluorescein,^29^ to a protein sample, photoexcitation can induce the formation of radical pairs between the above-mentioned aromatic amino acids and the exogenous electron acceptor, thus leading to photo-CIDNP hyperpolarization.^30,31^ Only solvent-exposed amino acids are capable of forming radical pairs since encounters between electron donor and acceptor are required for electron transfer.^32^ Additionally, the excited state of the electron acceptor must live sufficiently long to allow collisions with electron donor moieties.^33^ This is typically the case for dyes that undergo intersystem crossing into a triplet state after photoexcitation. Applications of this method include, but are not limited to, the investigation of the solvent accessibility of aromatic amino acids in proteins,^34,35^ determination of intramolecular correlation times and order parameters in proteins,^34,36^ and the investigation of folding processes on the millisecond time scale.^37^ While protein-bound flavin radicals have been observed in liquid-state NMR experiments using cysteine-devoid LOV domains with the photo-CIDNP method, reported cases are limited to intramolecular radical pairs.^38–40^ In this contribution, we investigate how liquid-state photo-CIDNP can be used to study solvent-exposed, protein-bound flavins.

In both riboflavin-LumP and Fld, the 7,8-dimethyl isoalloxazine core is solvent-exposed (see Figure S2). The surface accessibility of the flavin was determined by calculating the ratio of the solvent-accessible isoalloxazine moiety surface area in the flavin-protein complex to the whole isoalloxazine moiety surface area. The surface accessibility was found to be 0.34 and 0.20 in LumP and Fld, respectively. Similar to solvent-accessible amino acids in proteins, the photo-CIDNP efficiency depends on the square root of the total side chain accessibility of the respective amino acid.^35^ The ratio of the square roots of the solvent accessibilities of riboflavin-LumP and Fld is 1.30. Considering only solvent accessibility, no significant difference in the photo-CIDNP intensity would be expected between these two proteins.

Another relevant aspect of the formation of photo-CIDNP in solution is the lifetime of the excited state: in aqueous solutions, flavins are capable of forming spin-correlated radical pairs that can be detected by photo-CIDNP.^41^ In this case, the spin-correlated radical pairs are generated from a flavin triplet precursor.^42^ This is due to the short lifetime of the excited singlet state compared to that of the triplet, thus making bimolecular collisions between electron donors and acceptors more likely to occur in the triplet state.^33^

Photogenerated nuclear spin polarization can be observed after irradiation of a sample containing riboflavin-LumP and tryptophan (see Figure 1), since the protein-bound flavin forms a rather long-lived triplet state after photoexcitation with a lifetime of 3.1 μs for the excited and 13.1 μs for the relaxed triplet state at 279 K.^16^ In contrast, photoexcitation of Fld does not lead to a long-lived triplet state.^15^ The lifetime of the flavin’s excited state is therefore too short for radical-pair formation between the protein-bound flavin and tryptophan in solution, and hence, photo-CIDNP was not observed.

**Figure 1:**
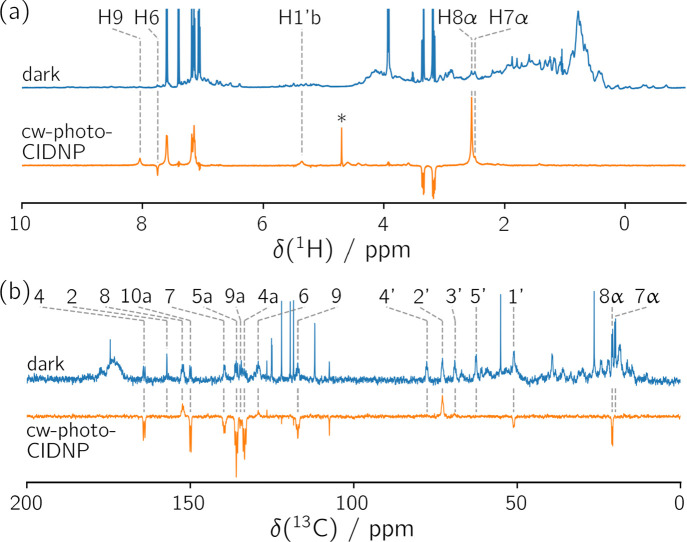
(a) Dark ^1^H NMR (blue, 64 scans) and ^1^H cw-photo-CIDNP spectra (orange, 64 scans) of riboflavin-LumP with exogenous tryptophan. The signal marked with an asterisk belongs to water, which was completely suppressed in the dark NMR spectrum using excitation sculpting.^43^ Flavin resonances from the xylene moiety and a proton from the ribityl side chain are labeled. The signal with a chemical shift of 5.36 ppm was assigned to the diastereotopic *pro-R* ^44^ proton (H1′b) attached to C1′ in the ribityl side chain.^45^ For signal assignment, see text. (b) Dark ^13^C NMR (blue, 8192 scans) and ^13^C cw-photo-CIDNP spectra (orange, 1024 scans) of [U-^13^C_17_]­riboflavin-LumP with exogenous tryptophan. The signal assignment is adapted from Illarionov et al.^46^ The sharp, unlabeled resonances in the dark NMR and cw-photo-CIDNP spectra belong to tryptophan and have been truncated in the dark NMR spectra.

The ^1^H NMR spectrum (panel a) acquired with a pulse sequence including water suppression (dark NMR spectrum) shows multiple signals originating from the numerous ^1^H nuclei present in riboflavin-LumP and the exogenous tryptophan, which was added in an approximately 25-fold excess. In contrast, the corresponding cw-photo-CIDNP spectrum specifically exhibits hyperpolarized ^1^H resonances from the protein-bound flavin and the tryptophan in solution. 64 scans are sufficient to observe the ^1^H nuclei in the protein-bound riboflavin when using solutions of approximately 200 μM riboflavin-LumP. Since the signal-to-noise ratio is quite good, the number of scans could be further reduced. Five flavin resonances from the aromatic protons H6 and H8, the methyl protons H7α and H8α and H1′b in the ribityl side chain could be assigned using tr-photo-CIDNP (see below). Three additional signals are observed in the cw-photo-CIDNP spectrum at 4.60, 4.43, and 3.60 ppm that do not arise from tryptophan. Based on their chemical shifts, these resonances presumably originate from protons in the ribityl side chain of riboflavin. Since they could not be detected in tr-photo-CIDNP experiments (see Figure S3), a definite assignment is not possible. The ^13^C resonances originating from [U-^13^C_17_]­riboflavin in riboflavin-LumP are easier to detect in the ^13^C dark NMR spectrum than the ^1^H resonances in the ^1^H dark NMR spectrum, see Figure 1b. The sharp resonances observed in the dark NMR as well as in the cw-photo-CIDNP spectrum belong to exogenous tryptophan. Signals of the quaternary ^13^C nuclei C4a, C5a and C10a show strong hyperpolarization. Besides large isotropic hyperfine coupling constants (see Table 2), low nuclear relaxation rates due to the absence of ^1^H nuclei in one-bond distance are responsible for their strong enhancement. Signal intensity and phase also strongly depend on secondary kinetics such as F-pair formation,^47^ degenerate electron exchange^48^ and dipolar cross-relaxation.^49^ In general, only nuclei with sufficiently large isotropic hyperfine coupling constants, or nuclei that are close to such, can be observed. This results in some nuclei not being detectable by photo-CIDNP, such as most ^1^H and ^13^C nuclei in the ribityl side chain of riboflavin.

The photo-CIDNP spectrum selectively exhibits resonances of ^1^H/^13^C nuclei in the moieties actively involved in electron transfer, i.e. protein-bound riboflavin and exogenous tryptophan. The method is nowadays routinely used to hyperpolarize solvent-exposed amino acids in proteins.^30,37,50^ Such an experiment is simple to perform and only requires the addition of FMN as photosensitizer prior to irradiation of the sample. The photo-CIDNP spectra in Figure 1 demonstrate that solvent-exposed flavins in flavoproteins can be studied just as easily by irradiating a mixture of a flavoprotein with an exogenous aromatic amino acid such as tryptophan. Changes in the photo-CIDNP resonances arising from the flavin can indicate changes in its electronic structure, in solvent accessibility and/or rate constants of photophysical processes such as the intersystem crossing rate.

Recording tr-photo-CIDNP data on freely diffusing radical pairs at high magnetic fields yields signal intensities that are proportional to the isotropic hyperfine coupling constant of the respective nucleus.^25,26^ In our setup, photoexcitation of the protein and the subsequent nuclear spin manipulation by RF pulses take place within a few microseconds, thus excluding secondary kinetics on the micro- to millisecond time scale that potentially distort this proportionality. The experiment thus provides valuable information about the electronic structure of the radicals involved based on hyperfine mapping. By comparing the tr-photo-CIDNP signal intensities with isotropic hyperfine coupling constants predicted by quantum chemical methods, such as density functional theory (DFT), the intermediate radical species present in the radical pair can be elucidated.

Figure 2a shows the tryptophan’s ^1^H signals measured by dark NMR (blue) and tr-photo-CIDNP (orange). In panel b, the signal intensities from the tr-photo-CIDNP experiment are plotted against the isotropic hyperfine coupling constants predicted using DFT for the cationic (top) and the neutral (bottom) tryptophan radical. The fact that the protonated cationic tryptophan radical is formed in pH-neutral solutions^51^ may be used to verify the validity of this method for the determination of the intermediate radical. Only ^1^H nuclei from the indole moiety of the tryptophan can be used because the hyperfine coupling constants of the protons bound to Cβ in the aliphatic chain depend on the dihedral angle between the Cβ–Hβ bond and the 2p_*z*_ orbital at C3, projected onto the plane perpendicular to the C3–Cβ bond.^52^ The determination of hyperfine coupling constants is therefore not possible from a single DFT calculation, but requires thermal averaging of multiple conformations with different dihedral angles. Figure 2b shows that the radical species can be determined, since a good correlation of signal intensities with DFT-calculated isotropic hyperfine coupling constants is obtained for the cationic tryptophan radical, while the correlation is worse for the neutral tryptophan radical. This demonstrates that this method yields reliable results even when a protein-bound flavin is used as electron acceptor.

**Figure 2:**
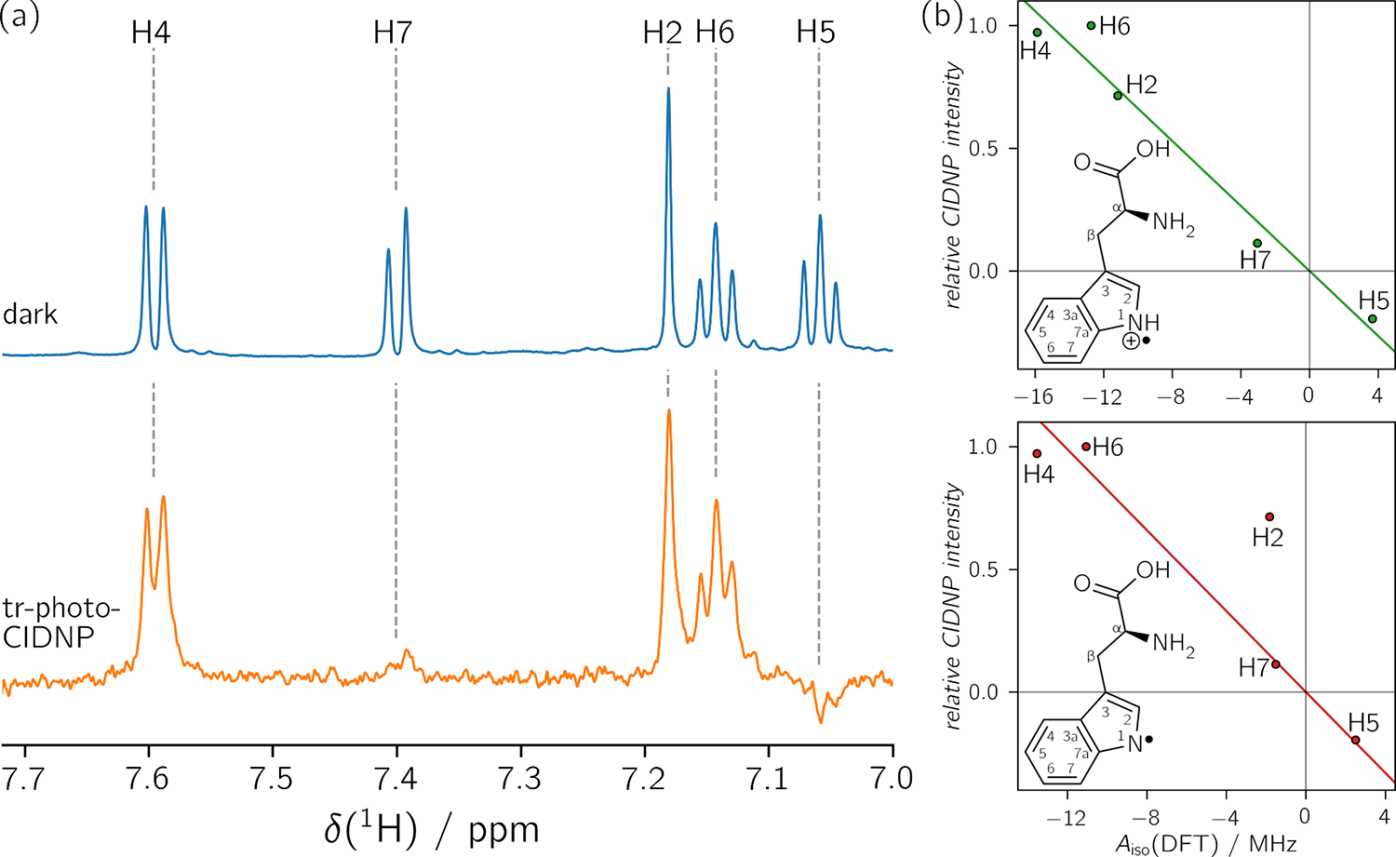
(a) Resonances of aromatic tryptophan protons in dark ^1^H NMR (blue, 16 scans) and ^1^H tr-photo-CIDNP spectra (orange, 1024 scans) of a sample containing riboflavin-LumP and tryptophan. The complete spectrum is shown in Figure S3. (b) Correlation of ^1^H tr-photo-CIDNP signal intensities with DFT-calculated isotropic hyperfine coupling constants of the cationic tryptophan radical (top, *R* ^2^ = 0.964, *m* = −0.0557 MHz^–1^) and the neutral tryptophan radical (bottom, *R* ^2^ = 0.698, *m* = −0.0694 MHz^–1^). Signal intensities were normalized with respect to the strongest signal observed, which was H8α of riboflavin (see Figure S4). The solid lines represent linear regressions forced to go through the origin. Experimentally obtained and DFT-calculated values for the isotropic hyperfine coupling constants are listed in Table S1. The signal assignment is adapted from Kiryutin et al.^51^

When comparing the DFT-calculated ^1^H hyperfine couplings of the two protonation states of the tryptophan radical, a striking find is that they may be distinguished based primarily on the data of H2, since all other protons show a similarly strong gain in isotropic hyperfine coupling upon protonation of the radical. This can be illustrated by correlating the DFT-calculated isotropic hyperfine coupling constants of the positively charged radical with those of the neutral radical (see Figure S5 and Figure 2b).

After confirming the proportionality relationship between tr-photo-CIDNP signal intensities and DFT-calculated hyperfine coupling constants for the tryptophan radical, the analysis can be extended to the riboflavin radical species present in the geminate radical pair by using photo-CIDNP data. In a pH-neutral aqueous solution, flavins form the anionic radical after electron transfer from tryptophan.^53,54^ By lowering the pH below the p*K*_a_ of the triplet precursor, the neutral flavin radical is formed. As the flavin in riboflavin-LumP is partially exposed to the solvent, we expect a similar pH dependence for the protein-bound riboflavin radical in the geminate radical pair.

To perform the analysis, a signal assignment is necessary for the ^1^H resonances of the protein-bound flavin. Those resonances are shown in Figure 3; for the complete ^1^H tr-photo-CIDNP spectrum, see Figure S3. Based on the chemical shifts, it is obvious that the two signals in the range between 7.7 and 8.1 ppm belong to the aromatic protons H6 and H9, and the two signals around 2.5 ppm to the methyl protons H8α and H7α. The signal at 5.36 ppm is assigned to a proton in the ribityl side chain. Tr-photo-CIDNP aids signal assignment since the signal intensity is proportional to the isotropic hyperfine coupling constant of a given nucleus at sufficiently high magnetic fields. Similar to the determination of the radical species, this information can be used when comparing known isotropic hyperfine coupling constants with photo-CIDNP signal intensities. In both neutral and anionic flavin radicals, H6 exhibits a strong (negative) isotropic hyperfine coupling constant, whereas H9 exhibits a small (positive) one.^53^ This is confirmed by DFT calculations using crystallographic data of riboflavin-LumP as input structure (see Table 1). The strong emissive signal in the aromatic region (7.75 ppm) of the tr-photo-CIDNP spectrum (see Figure 3a) is therefore assigned to H6 (*A*_iso_(DFT, anionic) = −9.39 MHz, *A*_iso_(DFT, neutral) = −4.43 MHz), while the weak absorptive signal (8.04 ppm) belongs to H9 (*A*_iso_(DFT, anionic) = 2.52 MHz, *A*_iso_(DFT, neutral) = 1.03 MHz). Using the same argument, the strong absorptive signal in the aliphatic region (2.55 ppm) can be assigned to H8α (*A*_iso_(DFT, anionic) = 11.99 MHz, *A*_iso_(DFT, neutral) = 7.81 MHz), and the weak emissive signal (2.49 ppm) to H7α (*A*_iso_(DFT, anionic) = −2.89 MHz, *A*_iso_(DFT, neutral) = 0.78 MHz). A further strong absorptive signal (5.36 ppm) is observed in the tr-photo-CIDNP spectrum; its chemical shift being too small to belong to the amide proton H3 suggests that it belongs to a ribityl side chain proton. DFT calculations predict only one large isotropic hyperfine coupling constant for ribityl side chain protons, which belongs to the proton H1′b (*A*_iso_(DFT, anionic) = 9.87 MHz, *A*_iso_(DFT, neutral) = 16.29 MHz). The signal assignment is summarized in Table 1.

**Figure 3:**
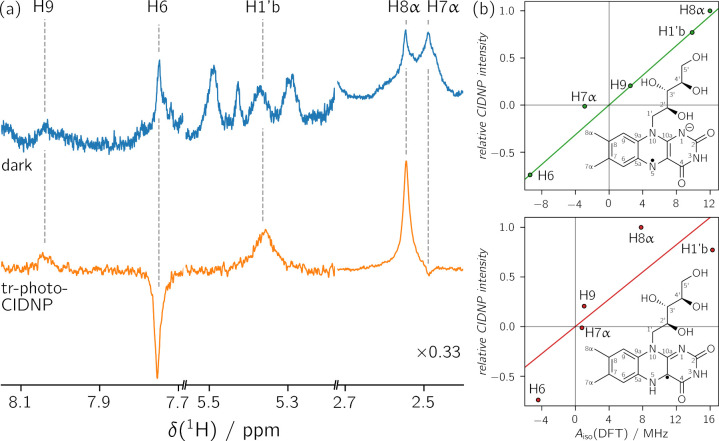
(a) Riboflavin resonances in dark ^1^H NMR (blue, 16 scans) and ^1^H tr-photo-CIDNP spectra (orange, 1024 scans) of a sample containing riboflavin-LumP and exogenous tryptophan. The complete spectrum is shown in Figure S3. The signals of H7α and H8α have been scaled by 0.33. (b) Correlation of tr-photo-CIDNP signal intensities with DFT-calculated isotropic hyperfine coupling constants of the anionic riboflavin radical (top, *R* ^2^ = 0.974, *m* = 0.0788 MHz^–1^) and the neutral riboflavin radical (bottom, *R* ^2^ = 0.709, *m* = 0.0686 MHz^–1^). The signal intensities of the methyl group protons were divided by 3. Additionally, signal intensities were normalized with respect to the highest value (H8α). The solid lines represent linear regressions forced to go through the origin. Experimentally obtained and DFT-calculated isotropic hyperfine coupling constants are listed in Table 1.

**Table 1:** Chemical Shifts and Isotropic ^1^H Hyperfine Coupling Constants for Protons of the Anionic and Neutral Riboflavin Radicals in Riboflavin-LumP^a^

			anion radical		neutral radical	
nucleus	δ/ppm	rel. *A*_iso_ (CIDNP)	rel. *A*_iso_ (DFT)	*A*_iso_/MHz (DFT)	rel. *A*_iso_ (DFT)	*A*_iso_/MHz (DFT)
H3	–	0.00	–0.04	–0.53	–0.25	–1.97
H6	7.75	–0.74	–0.78	–9.39	–0.57	–4.43
H7α	2.49	–0.01	–0.24	–2.89	0.10	0.78
H8α	2.55	1.00	1.00	11.99	1.00	7.81
H9	8.04	0.20	0.21	2.52	0.13	1.03
H1′a	–	0.00	0.10	1.16	0.17	1.35
H1′b	5.37	0.77	0.82	9.87	2.09	16.29
H2′	–	0.00	–0.05	–0.56	–0.11	–0.87

a Experimentally obtained and DFT-calculated isotropic hyperfine coupling constants were normalized with respect to H8α. Signals arising from H1′a, H2′, and H3 could not be measured.

The ribityl side chain of riboflavin is expected to contribute to the binding affinity of riboflavin to LumP, especially since the 7,8-dimethyl isoalloxazine moiety is exposed to the solvent. This is reflected by the fact that the binding affinity of DMRL to LumP largely depends on the stereoconfiguration of its ribityl side chain,^55^ thus suggesting that it assumes a rigid conformation. In such a case, the isotropic hyperfine coupling constants of the ribityl side chain protons obtained by DFT can be used without thermal averaging, since only one geometry is possible unlike in tryptophan where a rotation about C–C single bonds is possible in aqueous solution. The same applies to the ribityl side chain of free flavins in aqueous solution.

The unpaired electron-spin density in the xylene moiety of the riboflavin radical decreases upon protonation of N5. This is accompanied by a relatively uniform decrease of the isotropic ^1^H hyperfine coupling constants. A correlation of DFT-calculated isotropic hyperfine coupling constants for the anionic riboflavin radical against the neutral riboflavin radical is shown in Figure S6. The largest deviation is found for the methyl protons H7α. Unfortunately, the signal of these protons overlaps with the signal of the strongly polarized H8α nuclei and therefore cannot be determined precisely. For this reason, an unambiguous assignment of the present radical species based solely on ^1^H from the xylene moiety is not possible. The additional information provided by the H1′b proton is therefore very useful in distinguishing between the anionic and neutral flavin radical. When this information is added to the ^1^H correlation plot, the coefficients of determination increase from 0.968 to 0.974 for the anionic flavin radical and decrease from 0.973 to 0.709 for the neutral flavin radical (see Figure 3b and Figure S7). This indicates that the anionic riboflavin radical is present in the radical pair.

Since the photo-CIDNP signal intensities were normalized with regard to the same signal (H8α) for the correlation plots of the tryptophan (Figure 2) and the riboflavin radicals (Figure 3), the slopes of the correlation plots can be compared to analyze quenching of photo-CIDNP intensities. While the correlation is good when using all nuclei from the cationic tryptophan and anionic flavin radical (*R* ^2^ = 0.9503, see Figure S9), the slopes for the individual correlations differ (−*m*(TrpH^•+^) = 0.0557 MHz^–1^ and *m*(Rfl^•–^) = 0.0788 MHz^–1^), even after applying a factor of –1 to the slope of the cationic tryptophan radical correlation to account for Kaptein’s sign rule.^56^ This indicates that the photo-CIDNP intensity of the tryptophan radical (TrpH^•+^), which is initially protonated directly after electron transfer, is quenched. The rate of deprotonation to yield the neutral Trp^•^ from TrpH^•+^ is quite high in a pH-neutral solution (1.5 × 10^6^ s^–1^).^57^ However, the rate of degenerate electron exchange for the tryptophan radical in a pH-neutral solution was found to be 9 × 10^8^ M^–1^s^–1^.^58^ Applied to the present case with a tryptophan concentration of 5 mM this yields a photo-CIDNP decay rate by degenerate electron exchange of 4.5 × 10^6^ s^–1^, which is three times higher than the rate of deprotonation. Hence, degenerate electron exchange is the preferred mechanism by which the photo-CIDNP signal intensity of the tryptophan radical is quenched.

To further investigate the electronic structure of the radical and to validate the protonation state of the flavin radical in the geminate radical pair, ^13^C tr-photo-CIDNP experiments have been performed. Figure 4a shows the ^13^C dark NMR and ^13^C tr-photo-CIDNP spectra obtained for [U-^13^C_17_]­riboflavin-LumP with exogenous tryptophan. Two carbon atoms found in the 7,8-dimethyl isoalloxazine moiety show no signals in the tr-photo-CIDNP spectrum, namely C2 and C7α, since they exhibit only small isotropic hyperfine couplings (see Table 2). Their signal intensity in the tr-photo-CIDNP measurement was interpreted as 0. Additionally, the three carbon atoms C3′, C4′ and C5′ from the ribityl side chain were also not detected using tr-photo-CIDNP. This is expected because the unpaired electron-spin is mainly delocalized over the 7,8-dimethyl isoalloxazine core. Since these three atoms do not add any information, they were not included in the analysis.

**Table 2:** Isotropic Hyperfine Coupling Constants of ^13^C Nuclei in the Anionic and Neutral Riboflavin Radicals in [U-^13^C_17_]­riboflavin-LumP^a^

		anion radical		neutral radical	
nucleus	rel. *A*_iso_ (CIDNP)	rel. *A*_iso_ (DFT)	*A*_iso_/MHz (DFT)	rel. *A*_iso_ (DFT)	*A*_iso_/MHz (DFT)
C2	0.00	0.08	2.00	–0.07	–1.04
C4	–0.19	0.06	1.50	–0.48	–7.09
C4a	–0.65	–0.53	–13.08	0.35	5.15
C5a	–1.00	–1.00	–24.59	–0.92	–13.47
C6	0.18	0.45	10.95	0.22	3.22
C7	–0.40	–0.46	–11.27	–0.25	–3.62
C7α	0.00	0.05	1.27	–0.03	–0.51
C8	0.44	0.61	14.96	0.57	8.31
C8α	–0.27	–0.25	–6.18	–0.27	–3.90
C9	–0.49	–0.46	–11.23	–0.49	–7.17
C9a	0.04	0.30	7.31	0.05	0.67
C10a	–0.29	–0.22	–5.30	–1.00	–14.69
C1′	–0.12	–0.19	–4.73	–0.42	–6.24
C2′	0.39	0.30	7.30	0.57	8.33

a The isotropic hyperfine coupling constants were normalized with respect to C5a (experimental and DFT-calculated values for the anionic radical) and C10a (DFT-calculated values for the neutral radical). No signals were detected for C2, C7α as well as the ribityl side chain carbon atoms C3′, C4′ and C5′.

**Figure 4:**
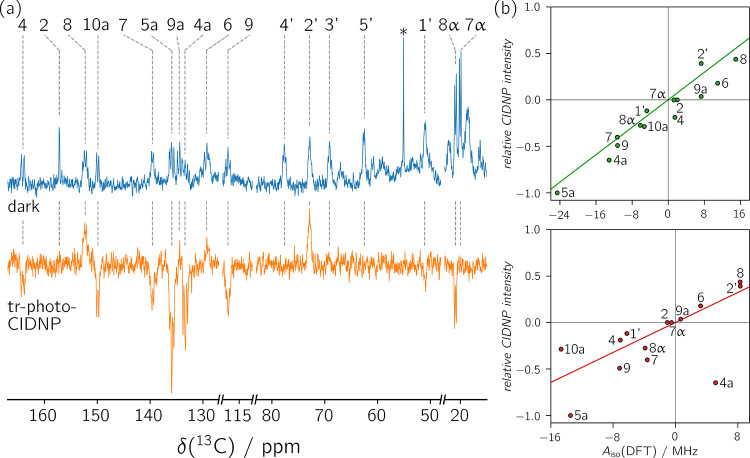
(a) Riboflavin resonances in dark ^13^C NMR (blue, 8192 scans) and ^13^C tr-photo-CIDNP spectra (orange, 2048 scans) of a sample containing [U-^13^C_17_]­riboflavin-LumP and exogenous tryptophan. The signal marked with an asterisk belongs to Cα of tryptophan. The complete spectrum is shown in Figure S4. (b) Correlation of tr-photo-CIDNP signal intensities with DFT-calculated isotropic hyperfine coupling constants of the anionic riboflavin radical (top, *R* ^2^ = 0.873) and the neutral riboflavin radical (bottom, *R* ^2^ = 0.414). Signal intensities were normalized with respect to the highest absolute value (C5a). Since signals arising from C2 and C7α could not be detected using tr-CIDNP, their signal intensities were interpreted as 0. The solid lines represent linear regressions forced to go through the origin. Experimentally obtained and DFT-calculated values for the isotropic hyperfine coupling constants are listed in Table 2. The structures of the riboflavin radicals are depicted in Figure 3b.

Correlation of ^13^C tr-photo-CIDNP signal intensities with DFT-calculated isotropic hyperfine coupling constants of the neutral and anionic radicals (Table 2) strongly favors the anionic radical as the radical occurring in the geminate radical pair (see Figure 4). This confirms the results obtained by ^1^H tr-photo-CIDNP measurements. Using ^13^C tr-photo-CIDNP, the distinction between the two possible radicals is even clearer (anionic: *R* ^2^ = 0.873, neutral: *R* ^2^ = 0.414), since more nuclei are available, including more nuclei from outside the xylene moiety.

To compare anionic flavin radicals in different environments, published relative hyperfine coupling constants of the anionic FMN radical in aqueous solution obtained with tr-photo-CIDNP^53,54^ were correlated with those obtained for riboflavin in LumP (see Figure 5). The correlation is very good for both ^1^H (*R* ^2^ = 0.994) and ^13^C (*R* ^2^ = 0.950) nuclei, indicating that there are only minor differences in the electronic structures of riboflavin bound to LumP and FMN in aqueous liquid solution. In riboflavin-LumP, the entire 7,8-dimethyl isoalloxazine core of the protein-bound riboflavin is solvent-exposed (see Figure S2a). While the pyrimidine moiety is slightly buried, the xylene moiety is strongly exposed. It is therefore not surprising that the correlation is particularly good for the xylene protons, as their environment is very similar in both aqueous as well as in the protein-bound flavin. For the ^13^C nuclei, isotropic hyperfine coupling constants of the nuclei in the xylene moiety, namely C6, C7, C7α, C8, C8α and C9, are very close to the ideal correlation (represented by the linear regression in Figure 5b), while the largest deviations are observed for C4a and C9a. However, errors in the integration can also be introduced by the overlap of signals from nuclei C4a, C5a, and C9a, which can cause the observed deviations.

**Figure 5:**
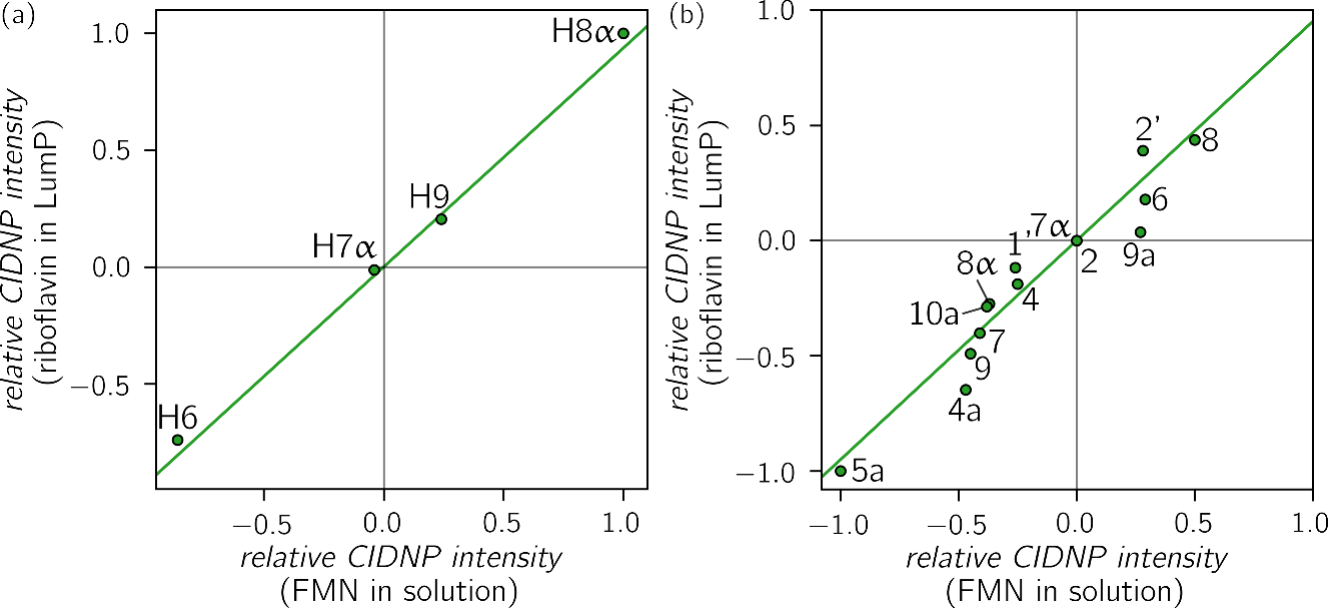
Correlation of tr-photo-CIDNP signal intensities of the anionic riboflavin radical in LumP with tr-photo-CIDNP signal intensities of the anionic FMN radical in liquid aqueous solution for ^1^H nuclei^53^ (a, *R* ^2^ = 0.994) and ^13^C nuclei^54^ (b, *R* ^2^ = 0.924). Signal intensities of methyl group protons were divided by 3. Additionally, signal intensities were normalized with respect to the strongest signal. The solid lines represent linear regressions forced to go through the origin.

Only limited data are available for hyperfine couplings of protein-bound flavins. Table 3 lists isotropic hyperfine coupling constants of the xylene protons in aqueous solution,^53^ in riboflavin-LumP and in glucose oxidase from *Aspergillus niger*.^59^ As discussed, the isotropic hyperfine coupling constants of these protons are very similar in riboflavin-LumP and in aqueous solution, while they are about 13% lower for H6 and H8α in glucose oxidase compared to riboflavin-LumP. This indicates that, in the case of glucose oxidase, the population of unpaired electron-spin is reduced in the xylene moiety due to interactions of the protein side chains with the 7,8-dimethyl isoalloxazine core.

**Table 3:** Isotropic Hyperfine Coupling Constants (in MHz) of Anionic Flavin Radicals in Aqueous Solution,^53^ Riboflavin-LumP, and *Glucose Oxidase* at pH 10 ^59^ ^a^

nucleus	aqueous solution	riboflavin-LumP	*glucose oxidase*
H6	–9.22	–9.39	(−)8.20
H7α	–3.19	–2.89	–
H8α	12.32	11.99	10.45
H9	2.86	2.52	–

a Isotropic hyperfine coupling constants of H7α and H9 are small and were not resolved.

In summary, photo-CIDNP is a powerful tool to selectively hyperpolarize the resonances of flavin cofactors bound to flavoproteins. For this method to be applicable to a given flavoprotein, two conditions must be met: first, the flavin cofactor of interest must be solvent-exposed, and second, a sufficiently long-lived excited state is required for radical-pair formation between the protein-bound flavin and the exogenous tryptophan. For strong signal enhancement, cw-photo-CIDNP is superior to tr-photo-CIDNP. However, tr-photo-CIDNP adds information that is potentially useful for signal assignment: the signal intensities at high magnetic fields are proportional to the isotropic hyperfine coupling constants of the respective nuclei in freely diffusing radical pairs.^25,26^ Comparison of tr-photo-CIDNP signal intensities with DFT predictions for isotropic hyperfine coupling constants not only allows assignment of the resonances, but also determination of the flavin radical present in the geminate radical pair, which was identified to be the anionic radical in LumP.

The anionic riboflavin radical has already been observed in the photocycle of riboflavin-LumP,^16^ albeit in small amounts. In this contribution, the electronic structure of this radical has been elucidated using photo-CIDNP. Compared to EPR spectroscopy, photo-CIDNP has two advantages: First, isotropic hyperfine coupling constants can be determined in aqueous solution and at room temperature (physiological conditions), and second, even small hyperfine coupling constants can be easily determined. These hyperfine coupling constants are influenced by the surrounding protein environment and are directly correlated to the electron-spin population of the respective nucleus, providing insight into the electronic structure of the flavin.^60^ They are therefore relevant parameters for understanding the reactivity of a given protein. It remains to be clarified how the anionic radical is formed in the absence of the electron donor tryptophan. One possible explanation is the formation of an intramolecular radical pair with an aromatic amino acid of the protein. Radical-pair formation is a key feature observed in several photoactive flavoproteins such as cryptochromes,^61^ photolyases^62,63^ and LOV domains.^38^ It is also possible that the small amount of anionic radical is a byproduct of unnaturally high (biologically irrelevant) concentrations of riboflavin-LumP. This could allow collisions between protein-bound riboflavin and solvent-exposed aromatic amino acids (e.g., tryptophan 186) from another riboflavin-LumP molecule, allowing radical-pair formation between them. As the biological relevance of riboflavin-LumP, which occurs naturally in *Photobacterium leiognathi*,^18^ is still unknown, further investigations into the role of the anionic radical are required.

## Experimental Methods

### Sample Preparation

Wild-type LumP from *Photobacterium leiognathi* and Fld from *E. coli* were expressed using protocols described elsewhere.^16,64^ [U-^13^C_17_]­Riboflavin was prepared from [U-^13^C_13_]­DMRL as described earlier.^46,65^ For incorporation of [U-^13^C_17_]­riboflavin into LumP, 200 μM [U-^13^C_17_]­riboflavin was incubated with 40 μM LumP, a mixed population protein loaded with either riboflavin or DMRL (both unlabeled), in 11.8 mM sodium/potassium phosphate, pH 7.4, containing 137 mM NaCl and 2.7 mM KCl (buffer A). After 1.5 h at room temperature in the dark, the solution was concentrated at 277 K to approximately 100 μL using a centrifugal filter (Amicon Ultra, Merck, Darmstadt, Germany; MWCO: 10 kDa) and then diluted to the initial volume with a solution of 200 μM [U-^13^C_17_]­riboflavin in buffer A. This process was repeated twice. After the final dilution step, the solution was kept at 277 K overnight. Excess cofactor was eluted by dilution with buffer A followed by concentration of the solution until the flow-through was colorless.

Photo-CIDNP samples contained 5 mM l-tryptophan (Sigma-Aldrich, St. Louis, MO, USA) in buffer A with either 100% D_2_O (99.95 atom-% D, Deutero GmbH, Kastellaun, Germany) for ^1^H photo-CIDNP measurements or 10% D_2_O for ^13^C photo-CIDNP measurements. The respective protein was added to a final absorbance of 2.5 at the respective absorption maximum: 461 nm (riboflavin-LumP) and 467 nm (Fld). Using the determined extinction coefficient for riboflavin-LumP of ϵ­(461 nm) = 11900 M^–1^cm^–1^ and a published extinction coefficient for Fld (ϵ­(467 nm) = 8250 M^–1^cm^–1^),^66^ an absorbance of 2.5 at the respective absorption maxima gives concentrations of 210 μM (riboflavin-LumP) and 300 μM (Fld).

### Determination of Riboflavin-LumP Extinction Coefficient

The extinction coefficient of riboflavin-LumP was determined using a slightly modified procedure described by McKean et al.^67^ Two equivalent samples containing riboflavin-LumP in buffer A were prepared. One sample was diluted by a factor of 0.5 with buffer A, while the second sample was unfolded by dilution by a factor of 0.5 with buffer A containing SDS (saturated). After incubation for 90 min at room temperature, the extinction coefficient was determined by comparing the absorbance of both samples and assuming ϵ­(461 nm) = 12500 M^–1^cm^–1^ for the riboflavin in buffer A containing SDS.^68^ The error was estimated by repeating the experiment twice. This method gave ϵ­(461 nm) = (11900 ± 400) M^–1^cm^–1^ for riboflavin-LumP.

### NMR Spectroscopy

NMR experiments were performed on an Avance III HD NMR spectrometer (Bruker BioSpin GmbH, Ettlingen, Germany) with a magnetic field strength of 14.1 T, resulting in resonance frequencies of 600 MHz for ^1^H and 151 MHz for ^13^C. ^1^H measurements were performed with a triple-resonance (TXI) probe optimized for proton observation, while ^13^C measurements were carried out with a broad-band (BBFO) probe. All measurements were performed at 293 K with sample volumes of 600 μL.

^1^H photo-CIDNP spectra were acquired using a presaturation pulse train designed for background suppression prior to optical excitation,^69^ including the solvent signal (HDO). A destructive phase cycle with irradiation in every second scan was used to acquire ^1^H and ^13^C photo-CIDNP spectra. In addition, a WALTZ-16 sequence was incorporated to decouple ^1^H in ^13^C photo-CIDNP spectra.^70^ Inverse-gated decoupling was chosen to minimize contributions from the nuclear Overhauser effect.^71,72^ A sampling pulse with a pulse length of 2.5 μs was used to acquire time-resolved (tr) ^1^H photo-CIDNP spectra, corresponding to a flip angle of 30°. The short pulse length ensured that contributions from secondary CIDNP processes such as F-pair formation^47^ and degenerate electron exchange^48^ were reduced. For ^13^C photo-CIDNP measurements, a sampling pulse length of 11 μs (corresponding to a flip angle of 90°) was necessary because the signal intensities of ^13^C photo-CIDNP are low compared to those of ^1^H photo-CIDNP. In all photo-CIDNP experiments, prescan delays (DE) of 6.5 μs and recycle delays (D1) of 5 s were used. For tr-photo-CIDNP measurements, light excitation was performed using a nanosecond-pulsed laser system, consisting of an OPO (OPO Plus, Continuum, Milpitas, CA, USA) pumped with a Nd:YAG laser (Surelite II, Continuum, Milpitas, CA, USA), resulting in a laser pulse length of 4–7 ns, output powers of 11–15 mJ and an illumination bandwidth (fwhm) of (2.2 ± 0.5) nm (see Figure S3). For tr-photo-CIDNP measurements, the samples were excited at their respective absorption maxima. For cw-photo-CIDNP measurements, samples were illuminated with a cw-laser (DHOM-H-445, Ultralasers, Newmarket, Canada) at 445 nm for 0.5 s prior to every second scan. The laser output was coupled into an optical fiber with a diameter of 1 mm (Thorlabs, Newton, NJ, USA) and inserted into the sample via a coaxial insert (Wilmad WGS-5BL).

Dark ^1^H NMR spectra were recorded using water suppression with excitation sculpting.^43^ Power gated decoupling using a WALTZ-16 scheme was used for dark ^13^C NMR spectra.^70^

### Quantum Mechanical Modelling

DFT calculations were performed using the ORCA program package (version 5.0.3).^73^ A microsolvation model was used as input structure for tryptophan (8Zpg+).^74^ The model for deprotonated tryptophan was generated using Avogadro (version 1.2.0)^75,76^ based on the microsolvated model. The model of the riboflavin binding site in LumP was generated from the crystal structure of riboflavin-LumP from *Photobacterium kishitanii* (PDB entry: 3A35).^77^ The positions of the riboflavin cofactor as well as residues V41, S48, L49, T50, D62, I63, D64, Q65 and A66 were adopted. In addition, seven crystal water molecules near the 7,8-dimethyl isoalloxazine core of riboflavin were used. The amino acids were modified as described elsewhere.^16^ Insertion of hydrogen atoms and modifications of the structure were performed using Avogadro (version 1.2.0).^75,76^ Geometry optimization was performed using the BP86 functional^78^ for the riboflavin radicals and the B3LYP functional^79,80^ for the tryptophan radicals. The def2-TZVP basis set was used for all geometry optimizations.^81^ The geometry of the 7,8-dimethyl isoalloxazine moiety in the riboflavin and all inserted hydrogen atoms were optimized, while all non-hydrogen atoms of the residues and the oxygen atoms of the crystal water were left in their crystallographic positions. The optimized structures of the anionic and neutral riboflavin radicals in LumP are shown in Figure S1. Electronic parameters were calculated using the B3LYP functional^79,80^ in conjunction with the EPR-II basis set.^82^ In all calculations, def2/J was chosen as the auxiliary basis.^83^ An atom-pairwise dispersion correction was applied to account for dispersion forces.^84,85^

### Surface Accessibility

Surface accessibility was calculated using PyMOL (version 2.5.4).^86^ Hydrogen atoms were inserted into the crystal structures of riboflavin-LumP from *Photobacterium kishitanii* (pdb entry: 3A35) and Fld from *E. coli* (pdb entry: 1AHN) at the appropriate positions, and the flavins were truncated at N10. Dots were generated for the solvent-accessible surface (set dot_solvent, 1) and the sampling rate was increased (set dot_density, 3). Surface accessibility was then calculated using the get_area command for the flavin in the flavin-protein complex and for the free flavin.

## Supplementary Material



## Notes

LumP models used for calculation of electronic parameters; surface representations; spectra of the pulsed laser that was used; additional photo-CIDNP data and correlation plots; DFT-calculated hyperfine coupling constants of aqueous tryptophan (PDF)
