# Supplementary material for: Photo-CIDNP of Solvent-Exposed Flavins in Flavoproteins
Source: J Phys Chem Lett. 2025 Jul 2;16(27):7058–68. doi: 10.1021/acs.jpclett.5c01239 (PMC12257599; doi:10.1021/acs.jpclett.5c01239)
Supplement: Supplementary file 1 [file jz5c01239_si_001.pdf]

Supporting Information

Photo-CIDNP of solvent-exposed flavins in flavoproteins

Anton Schmidt,[1] Hannah Schneider,[1] Boris Illarionov,[2] Adelbert Bacher,[3] Markus Fischer,[2] Stefan Weber[1]

[1] *Institute of Physical Chemistry, University of Freiburg, 79104 Freiburg, Germany*

[2] *Hamburg School of Food Science, Institute of Food Chemistry, University of Hamburg, 20146 Hamburg, Germany*

[3] *Department of Chemistry, Technical University of Munich, 85748 Garching, Germany*

Contents

I. DFT-optimized structures 1

II. Surface representations 1

III. Pulsed laser spectra 2

IV. Photo-CIDNP spectra 3

V. Correlation plots 5

VI. Tryptophan isotropic hyperfine coupling constants 7

VII. References 8

I. DFT-optimized structures

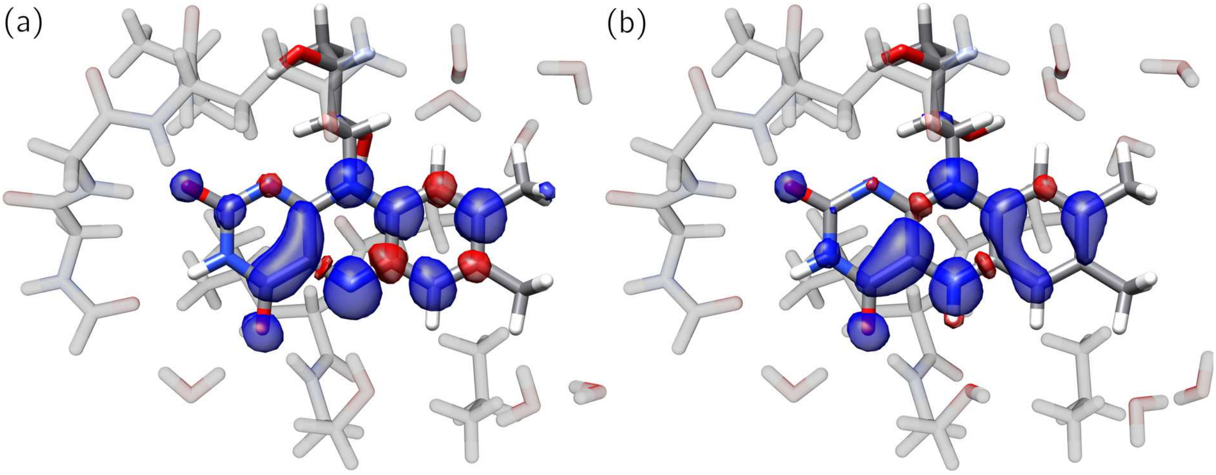

Figure S1: Models used for the calculation of electronic parameters for the riboflavin anionic radical (a) and neutral radical (b) bound by LumP. The residues and water molecules are displayed transparently. The spin density maps obtained via DFT are colored in blue (positive values) and red (negative values, contour 0.0025 a.u.). Atoms are colored in white (H), grey (C), blue (N) and red (O). Spin density plots were generated using the UCSF Chimera software.[1]

II. Surface representations

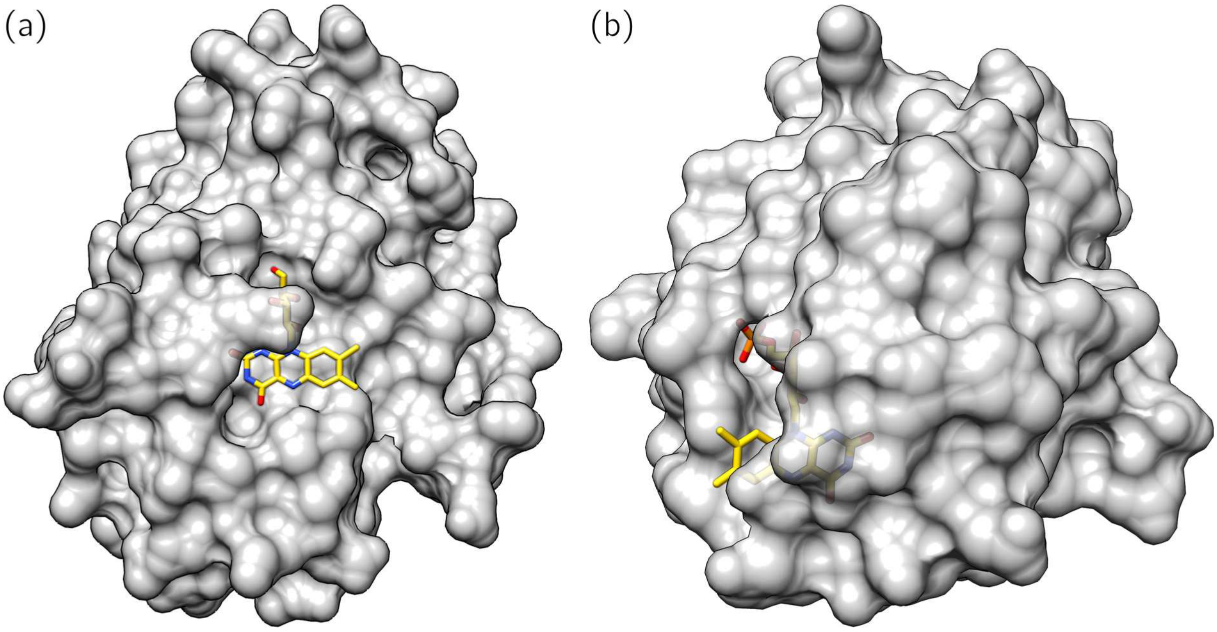

Figure S2: Surface plots of (a) lumazine protein (pdb entry: 3A35)[2] *and (b) flavodoxin (pdb entry: 1AHN).*[3] *Atoms of the flavin are colored in yellow (C), blue (N), red (O) and orange (P). The plot was generated using the UCSF Chimera software.*[1]

III. Pulsed laser spectra

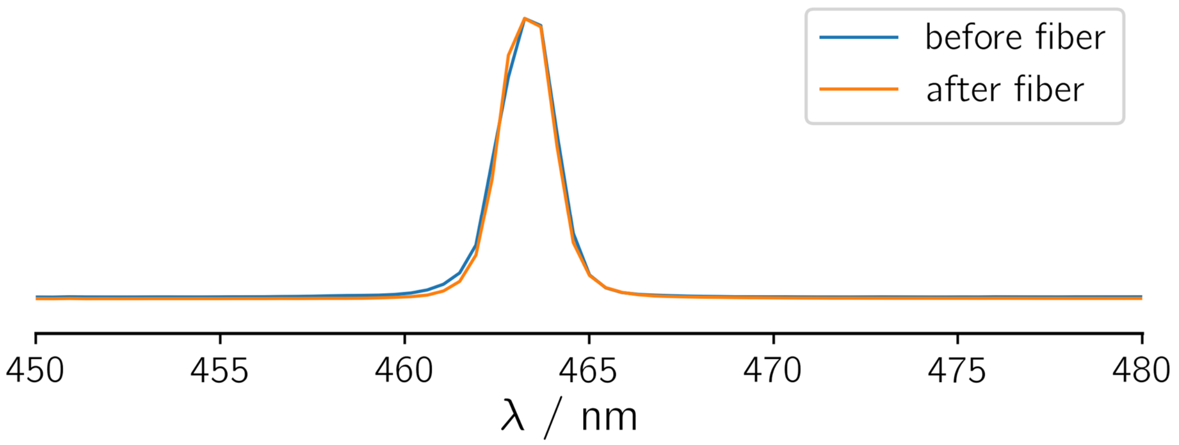

Figure S3: Spectrum of the pulsed laser system before (orange) and after (blue) coupling the output into an optical fiber. The OPO wavelength was set to 463 nm. No changes of the spectrum were observed.

IV. Photo-CIDNP spectra

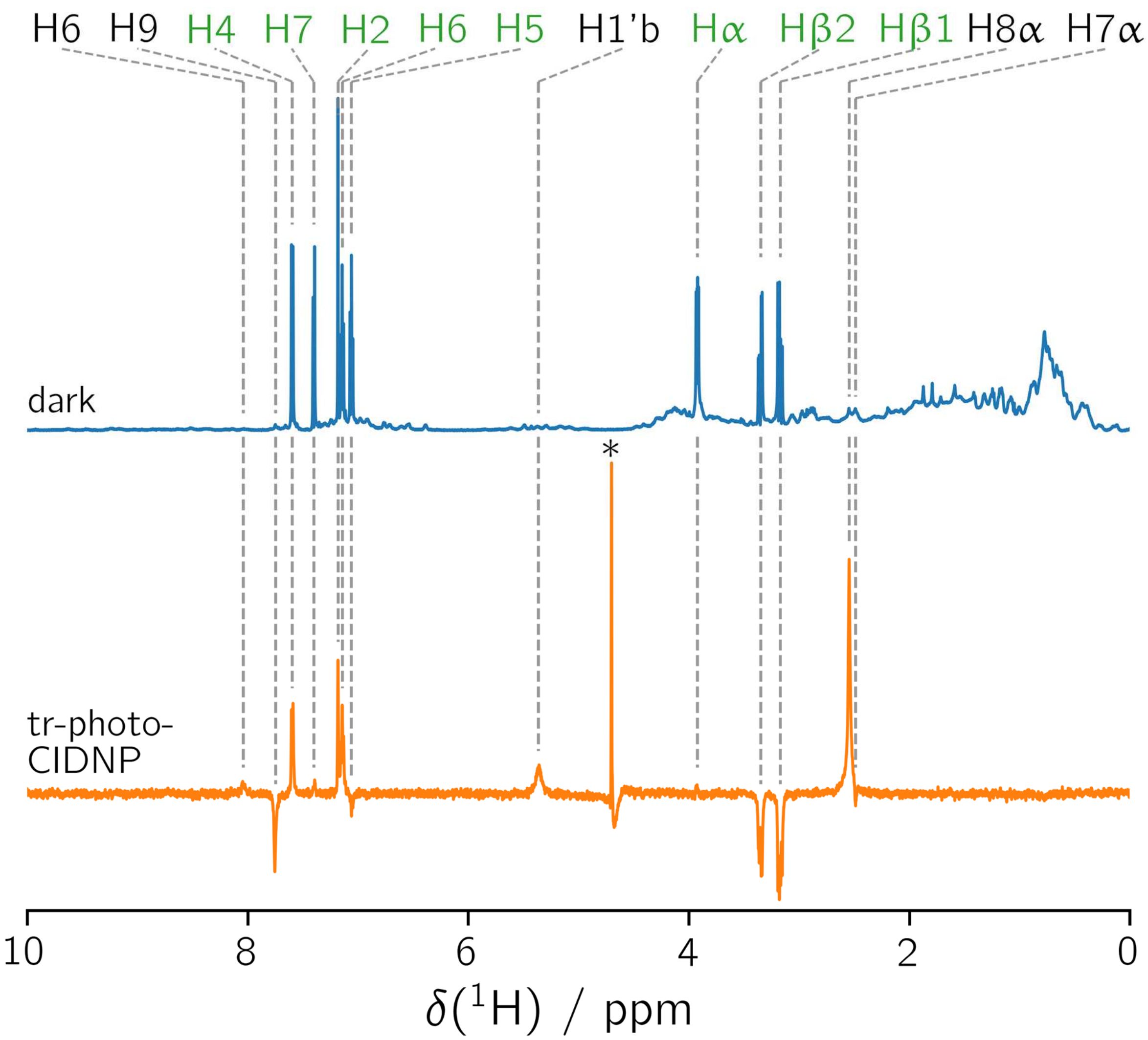

Figure S4: Riboflavin and tryptophan resonances in dark ^{1}H NMR (blue, 16 scans) and ^{1}H tr-photo-CIDNP (orange, 1024 scans) of riboflavin-LumP with exogenous tryptophan. The signal marked with an asterisk belongs to water, which was completely suppressed in the dark NMR spectrum using excitation sculpting.[4] Flavin resonances from the xylene moiety and a proton from the ribityl side chain are labeled. For better distinguishability, the protons of tryptophan are labeled in green.

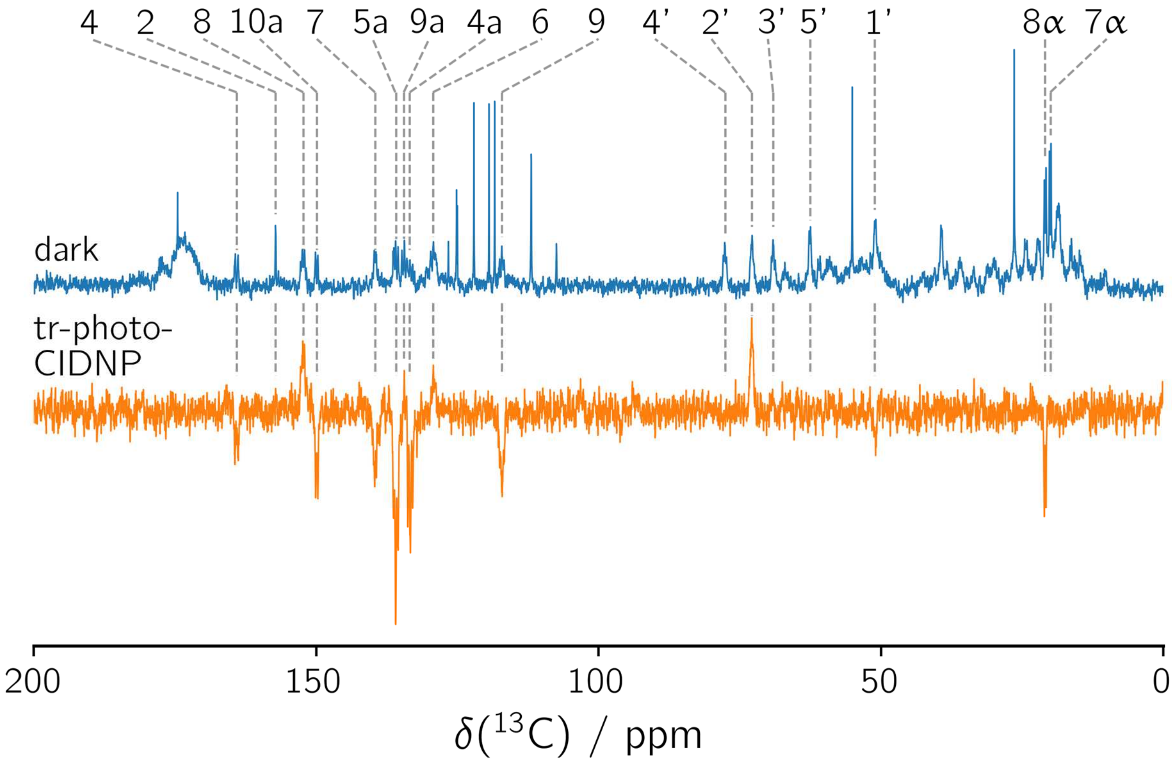

Figure S5: Riboflavin resonances in dark ^{13}C NMR (blue, 8192 scans) and ^{13}C tr-photo-CIDNP (orange, 2048 scans) of [U-$^{13}C_{17}$]riboflavin-LumP with exogenous tryptophan. All resonances from carbon atoms of the [U-^{13}C]riboflavin are labeled. The sharp, unlabeled resonances in the dark NMR spectrum belong to tryptophan.

V. Correlation plots

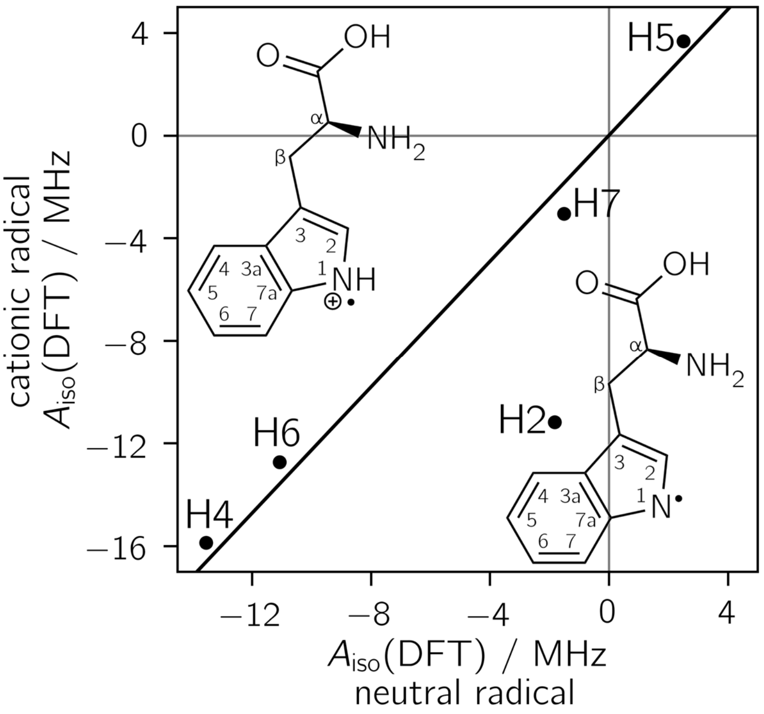

Figure S6: Correlation of DFT-calculated isotropic hyperfine coupling constants of the cationic tryptophan radical (top left) against the neutral tryptophan radical (bottom right).

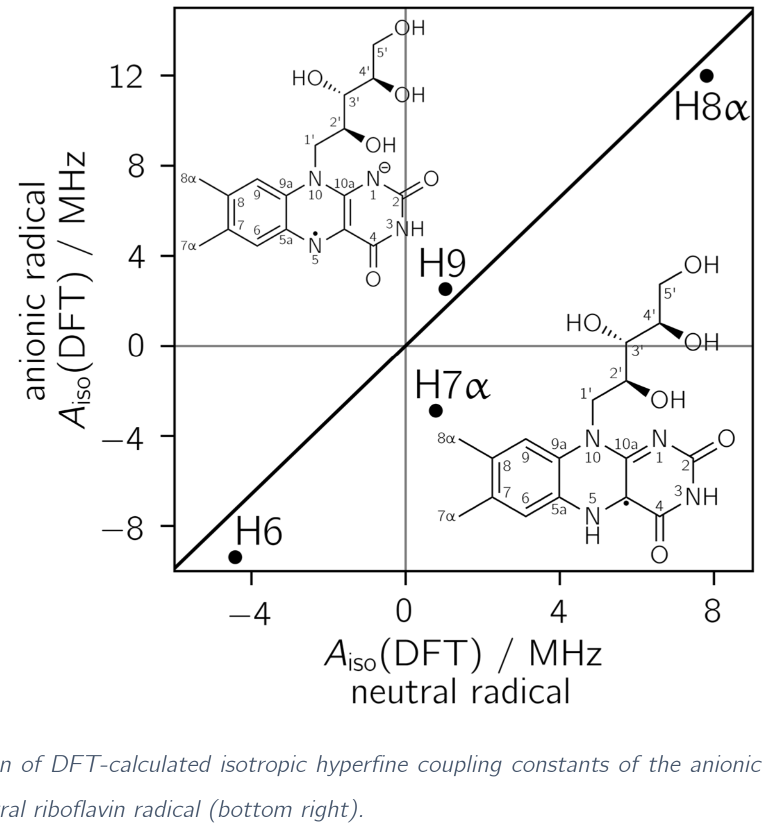

Figure S7: Correlation of DFT-calculated isotropic hyperfine coupling constants of the anionic riboflavin radical (top left) against the neutral riboflavin radical (bottom right).

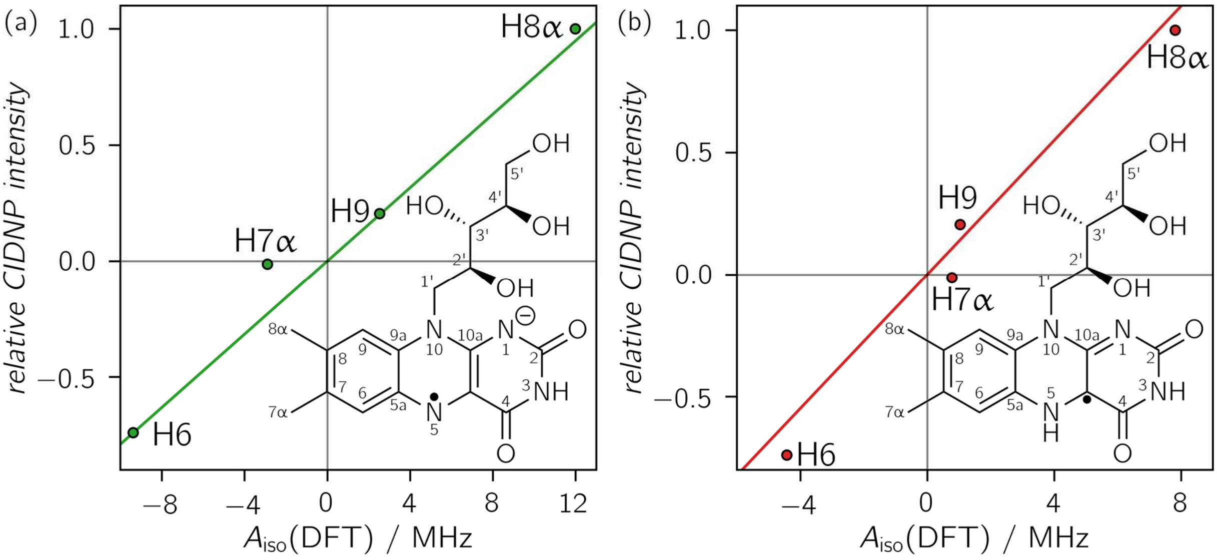

Figure S8: Correlation of tr-photo-CIDNP signal intensities with DFT-calculated isotropic hyperfine coupling constants of the anionic riboflavin radical (a, R^2 = 0.968) and the neutral riboflavin radical (b, R^2 = 0.973). The nucleus H1'b was excluded.

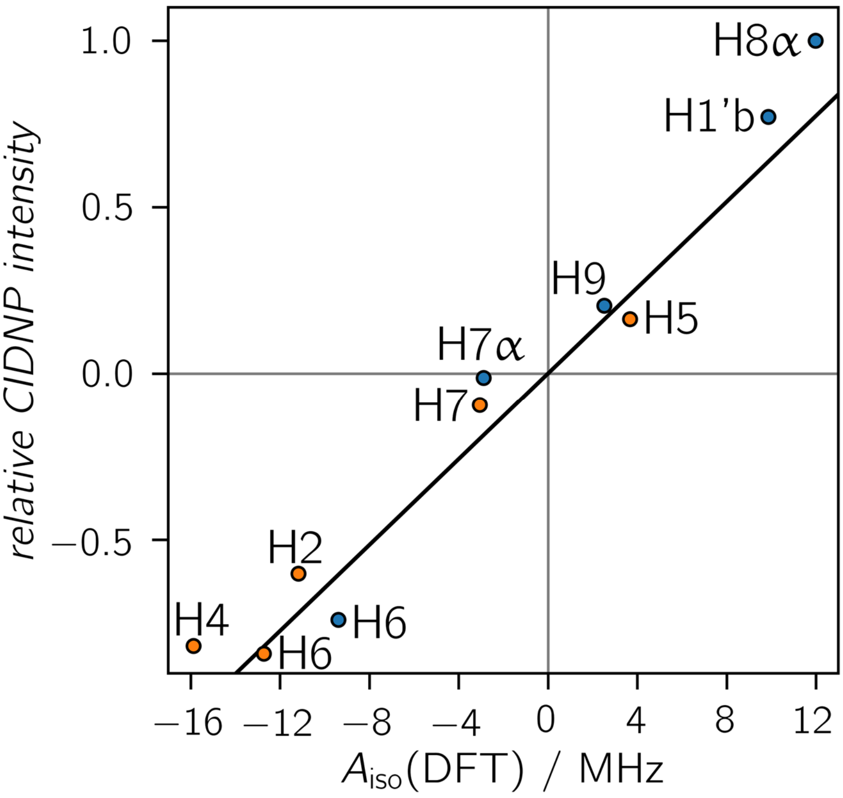

Figure S9: Correlation of tr-photo-CIDNP signal intensities with DFT-calculated isotropic hyperfine coupling constants (R^2 = 0.9503, m = 0.0645 MHz^{-1}) of the anionic riboflavin radical (blue circles) and cationic tryptophan radical (orange circles). The signal intensities of the methyl group protons were divided by 3. Additionally, signal intensities were normalized with respect to the highest value (H8α). The solid lines represent linear regressions forced to go through the origin.

VI. Tryptophan isotropic hyperfine coupling constants

Table S1: Isotropic hyperfine coupling constants for ^1H nuclei of the cationic and neutral tryptophan radicals. Experimentally obtained isotropic hyperfine coupling constants were normalized with respect to the strongest signal (H6), DFT-calculated isotropic hyperfine coupling constants were normalized with respect to the highest predicted value (H4).

		cationic radical		neutral radical	
Nucleus	rel. A_{iso} (CIDNP)	rel. A_{iso} (DFT)	A_{iso}/MHz (DFT)	rel. A_{iso} (DFT)	A_{iso}/MHz (DFT)
H2	0.71	0.70	−11.18	0.13	−1.82
H4	0.97	1	−15.87	1	−13.54
H5	−0.19	−0.23	3.67	−0.18	2.50
H6	1	0.80	−12.73	0.82	−11.07
H7	0.11	0.19	−3.05	0.11	−1.51

VII. References

1. E. F. Pettersen, T. D. Goddard, C. C. Huang, G. S. Couch, D. M. Greenblatt, E. C. Meng and T. E. Ferrin, "UCSF Chimera–a visualization system for exploratory research and analysis", *J. Comput. Chem.*, 2004, **25**, 1605–1612.
2. Y. Sato, S. Shimizu, A. Ohtaki, K. Noguchi, H. Miyatake, N. Dohmae, S. Sasaki, M. Odaka and M. Yohda, "Crystal structures of the lumazine protein from *Photobacterium kishitanii* in complexes with the authentic chromophore, 6,7-dimethyl-8-(1'-D-ribityl) lumazine, and its analogues, riboflavin and flavin mononucleotide, at high resolution", *J. Bacteriol.*, 2010, **192**, 127–133.
3. D. M. Hoover and M. L. Ludwig, "A flavodoxin that is required for enzyme activation: the structure of oxidized flavodoxin from Escherichia coli at 1.8 A resolution", *Protein Sci.*, 1997, **6**, 2525–2537.
4. T. L. Hwang and A. J. Shaka, "Water suppression that works. Excitation sculpting using arbitrary wave-forms and pulsed-field gradients", *J. Magn. Reson. Ser. A*, 1995, **112**, 275–279.
